# The Liver Tumor Microenvironment in Hepatocellular Carcinoma: Comparisons with Intrahepatic Cholangiocarcinoma and Therapeutic Implications

**DOI:** 10.3390/cancers18162696

**Published:** 2026-08-20

**Authors:** Kizuki Yuza, Jun Kawashima, Miho Akabane, Timothy M. Pawlik

**Affiliations:** 1 Department of Surgery, The Ohio State University Wexner Medical Center and James Comprehensive Cancer Center, Columbus, OH 43210, USA; kizuki.yuza@osumc.edu (K.Y.); miho.akabane@osumc.edu (M.A.); 2 Department of Gastroenterological Surgery, Yokohama City University, Yokohama 236-0004, Japan; kawashima.jun.da@yokohama-cu.ac.jp

**Keywords:** liver tumor microenvironment, hepatocellular carcinoma, intrahepatic cholangiocarcinoma, immune evasion, onco-fetal reprogramming, metabolic reprogramming, gut–liver axis, immune checkpoint inhibitors

## Abstract

The liver is an immunologically distinctive organ. Portal blood continuously delivers gut-derived antigens and microbial products, requiring hepatic immunity to balance surveillance with restraint. In hepatocellular carcinoma (HCC), this physiology is commonly overlaid by chronic injury, inflammation, and fibrosis, so the background liver forms part of the disease context in which the tumor microenvironment (TME) develops. This review synthesizes how cellular architecture, tumor-intrinsic programs, and structural, metabolic, and microbial conditions interact to shape immune evasion and heterogeneity. HCC provides the principal evidence base, with intrahepatic cholangiocarcinoma (iCCA) used as a structured, biologically distinct comparator. We organize therapies by the microenvironmental barriers they are intended to modify and distinguish established clinical efficacy from evidence that the proposed mechanisms mediate treatment benefit. Single-cell and spatial studies have resolved cellular states and spatial arrangements, including onco-fetal endothelial–myeloid neighborhoods and a macrophage–fibroblast boundary band separating lymphocyte-rich stroma from malignant tissue. These patterns operate within fibrotic and metabolically altered tissue and vary by etiology, spatial context, and tumor type. Vascular endothelial growth factor blockade with immune checkpoint inhibition and dual checkpoint blockade are established first-line options in advanced HCC. Chemo-immunotherapy is established in biliary tract cancer, and IDH1 inhibition has established efficacy in IDH1-mutant cholangiocarcinoma. Myeloid- and stroma-directed strategies, natural-product approaches, and engineered-cell therapies remain preclinical or early clinical. None of the pivotal trials tested whether the proposed microenvironmental mechanism mediated treatment benefit. The liver TME informs treatment selection without yet determining it.

## Simple Summary

Liver cancers grow in a complex environment of immune cells, blood vessels, supporting tissue, and metabolic signals. Treatment has advanced, but it is unclear which features of this environment explain that benefit or could guide treatment choice. This review focuses on hepatocellular carcinoma, the most common primary liver cancer, and uses intrahepatic cholangiocarcinoma, a bile duct cancer, as a biologically distinct comparison. We explain how tumors use their surroundings to suppress immune cells, keep them out, or weaken those that get in, and how chronic liver disease, tumor genetics, metabolism, and gut-derived signals make patients differ. Immune checkpoint-based treatments prolong survival in advanced hepatocellular carcinoma, while adding immunotherapy to chemotherapy prolongs survival in biliary tract cancer. Treatments aimed at supporting cells or scar tissue remain experimental. Distinguishing established findings from proposed explanations clarifies how the environment informs treatment and where further studies are needed.

## 1. Introduction

Primary liver cancer develops within an organ already responsible for filtering portal blood, regulating systemic metabolism, and balancing immune surveillance with tolerance. The clinical burden remains substantial. With an estimated 843,000 new cases and 732,000 deaths in 2024, liver cancer ranked seventh in incidence but third in cancer mortality worldwide [1]. Hepatocellular carcinoma (HCC) accounts for most primary liver cancers and provides the principal mechanistic and clinical evidence base for this review, whereas intrahepatic cholangiocarcinoma (iCCA), the second most common primary hepatic malignancy, is biologically distinct and marked by prominent stromal remodeling [2,3]. Their shared location within the liver does not imply a shared pathogenesis or microenvironment.

The liver’s dual blood supply converges in a low-pressure, fenestrated sinusoidal network through which portal inflow continuously delivers nutrients, gut-derived antigens, and microbial products, while gradients in oxygen and nutrient availability establish metabolic zonation across the lobule [4,5]. Because much of this portal input is harmless, hepatic immunity must couple rapid surveillance and clearance with restraint and is therefore better understood as a regulated balance than as constitutive immunosuppression [4]. These functions of the liver are features of normal physiology, but chronic injury and malignancy can reprogram the hepatic milieu. The tumor microenvironment (TME) that emerges is therefore best understood not as a fixed inventory of cells but as a network of interactions among tumor, immune, stromal, vascular, and metabolic compartments.

In HCC, the physiological baseline is frequently overlaid by chronic liver injury. Repeated hepatocyte damage, inflammation, regeneration, and fibrogenesis progressively alter immune regulation, vascular function, and tissue structure. Most HCC occurs in chronic liver disease—commonly, though not invariably, in the setting of cirrhosis [2]. The background liver is thus part of the disease context, shaping tumor initiation and progression as well as hepatic reserve and the capacity to tolerate treatment. In contrast, iCCA frequently arises without cirrhosis, although selected biliary and chronic liver disorders provide injury-associated contexts, and, once established, iCCA commonly contains an extensive desmoplastic stroma [3].

This review is centered on HCC, which provides most of the mechanistic and clinical evidence in this field. iCCA is examined as a structured, biologically distinct comparator, and combined hepatocellular-cholangiocarcinoma is beyond the scope of the review. We first describe the cellular architecture of the liver TME and the structural, metabolic, and microbial conditions that shape it. We then examine mechanisms of immune evasion and microenvironmental heterogeneity, highlighting where HCC and iCCA converge and where they diverge. Finally, we organize therapeutic strategies by the barriers they are intended to modify and distinguish demonstrated clinical efficacy from evidence that a proposed microenvironmental mechanism mediates treatment benefit. This distinction defines the maturity of current translational evidence.

This narrative review provides a targeted synthesis rather than a systematic review or meta-analysis; no formal risk-of-bias assessment or quantitative synthesis was performed. The literature base was updated through structured PubMed searches of English-language records conducted on 10 and 11 August 2026 using prespecified strings that combined HCC and iCCA terms with topic blocks covering cellular and spatial architecture, immune populations, tumor-intrinsic programs, metabolic and stromal pathways, systemic and emerging therapies, and natural products. Complete search strings, coverage intervals, and record counts for each module are provided in Supplementary Tables S1 and S2, and the screening, eligibility, and selection procedures are set out in the Supplementary Methods. Most modules covered 1 January 2016 through the relevant search date; those addressing nicotinamide N-methyltransferase and biliary tract chemo-immunotherapy, and any search by trial name, had no lower date boundary.

After duplicate removal by PMID, DOI, and title, all unique records in the first module were screened by title and abstract, while records in the remaining modules were ranked by topic relevance and screened from the highest-ranked downward, with the abstract consulted where the title alone was insufficient; potentially eligible reports were then assessed against the inclusion and exclusion criteria. Studies were eligible when they used an interpretable design and reported a result relevant to a prespecified topic in HCC or iCCA. Evidence from other tumor types was eligible only where it supplied a directly relevant mechanism or a narrowly bounded therapeutic rationale, and its non-hepatic context was stated explicitly wherever it is cited. Case reports, non-English reports, unvalidated in silico predictions, and correlation-only analyses without mechanistic or translational interpretation were excluded. Selection was purposive rather than exhaustive, consistent with a narrative review. Records that could not be resolved at screening were carried forward to full-text assessment rather than excluded. Reference lists of included studies and reviewer-nominated studies were assessed using the same criteria. Conference abstracts were considered only for relevant trials without a peer-reviewed full publication; accordingly, EMERALD-3 is identified throughout as a conference abstract and was verified separately from the PubMed search.

## 2. Cellular Architecture of the Liver Tumor Microenvironment

**Figure 1:**
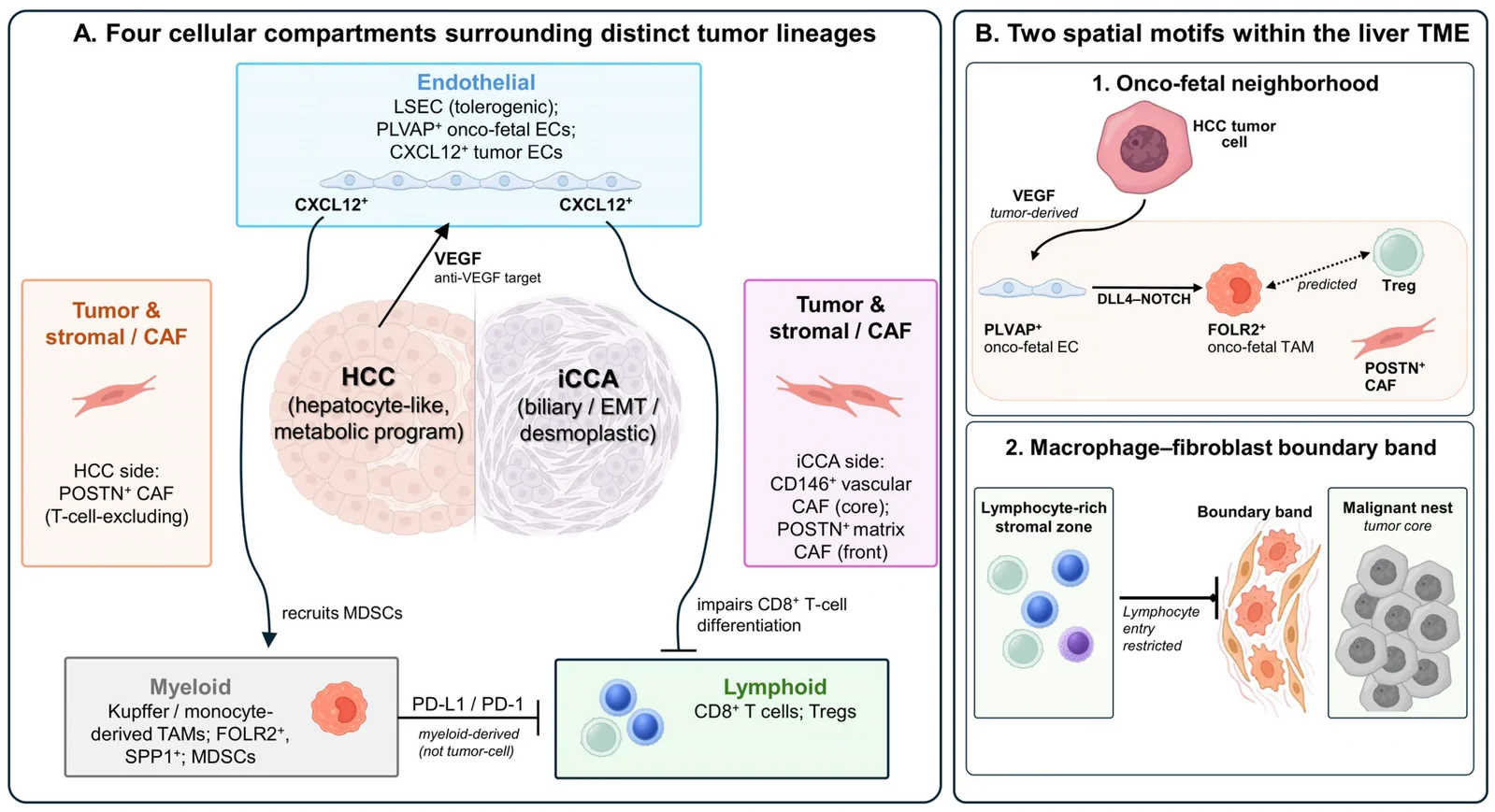
Cellular architecture and spatial organization of the liver tumor microenvironment. (**A**) Four cellular compartments—tumor and stromal, endothelial, myeloid, and lymphoid—shown around hepatocellular carcinoma (HCC) and intrahepatic cholangiocarcinoma (iCCA) cells, with lineage-associated fibroblast states on their respective sides. Representative cross-compartment interactions are shown. (**B**) Two recurrent spatial motifs: an onco-fetal endothelial–myeloid–regulatory neighborhood, a program also present in cirrhotic and regenerating liver, and a macrophage–fibroblast boundary band separating lymphocyte-rich stroma from malignant tissue. Arrowheads denote promoting or recruiting interactions, bar-heads inhibitory ones, and dashed lines associations inferred from spatial data alone. Compartments are distinguished by shape and label as well as by color.

The cellular compartment of the liver TME can be organized into four interacting groups: tumor and stromal cells, endothelial cells, myeloid cells, and lymphoid cells. Single-cell and spatial studies reveal distinct cellular states and recurrent spatial patterns within HCC and iCCA [6]. Less frequently profiled populations, including dendritic cells, neutrophils, natural killer (NK) cells, natural killer T (NKT) cells, mucosal-associated invariant T (MAIT) cells, γδ T cells, and B cells, are considered separately below. This section describes the principal populations and their interactions. The physical and metabolic conditions that shape them and the integrated mechanisms of immune escape are addressed in Section 3 and Section 4. Representative compartments and spatial motifs are summarized in Figure 1.

### 2.1. Malignant and Stromal Cells

Malignant cells generally retain lineage-associated transcriptional programs. HCC cells express hepatocyte and metabolic profiles, whereas iCCA cells display biliary epithelial and epithelial-to-mesenchymal transition programs [6]. The stromal compartment differs between the diseases in composition and spatial organization. In HCC, spatial multi-omic profiling identified fibroblasts co-expressing *COL1A2*, *COL4A1*, *COL4A2*, *CTGF*, and *FSTL1* within and around tumor nests, co-localized with malignant cells of higher stemness scores. Their abundance peaked at the tumor–liver interface and declined with distance toward both the tumor core and the adjacent liver, a gradient measured independently by transcriptomic score and by protein staining [7]. Higher density was associated with shorter overall survival in an independent 92-patient imaging cohort, and among collagen-expressing fibroblasts this association was specific to that state [7]. A separate FAP^+^ fibroblast-rich, immune-poor state has been described in a subset of HCC and was sustained partly by tumor-derived secreted phosphoprotein 1 (SPP1) signaling to fibroblasts [8]. In exploratory cohorts, POSTN^+^ cancer-associated fibroblasts (CAFs) marked T-cell-excluding regions and were associated with poorer response to immune checkpoint blockade when found with macrophages expressing SPP1 [9]. The spatial architecture of this association is discussed in Section 4.3.

Stromal tissue is particularly abundant in iCCA, although matrix amount, fibroblast activation, and collagen composition are distinct properties. CAFs are also heterogeneous. In experimental iCCA, lineage tracing identified hepatic stellate cells as the predominant CAF source [10]. Inflammatory CAFs promoted tumor growth through paracrine HGF-MET signaling, whereas myofibroblastic CAFs depended on hyaluronan synthase 2-mediated hyaluronan production [10]. Single-cell profiling of human iCCA further resolved a CD146^+^ vascular state in the tumor core and microvasculature and a periostin-expressing matrix state at the invasive front within collagen-rich streaks [11]. Selective deletion of CAF-derived type I collagen reduced matrix stiffness while tumor growth continued, illustrating that stiffness and tumor-promoting activity are separable [10]. A high pan-CAF signature was associated with shorter survival and increased recurrence in iCCA [10]. Marker-based CAF state names differ across studies, so the states are described here by both marker and position.

### 2.2. Endothelial Cells and Onco-Fetal Reprogramming

Endothelial cells in HCC are not merely vascular conduits; they can acquire states that organize local immune and stromal interactions. Liver sinusoidal endothelial cells (LSECs) normally form a fenestrated lining that permits solute exchange and contributes to hepatic immune tolerance, whereas chronic injury causes capillarization and loss of fenestrae [5]. In HCC, tumor-associated endothelial cells expressing *PLVAP* share transcriptional features with fetal liver endothelium and are enriched relative to adjacent liver [12]. Vascular endothelial growth factor (VEGF) supports this state, while endothelial DLL4–NOTCH signaling can drive monocytes toward a fetal-like FOLR2^+^ macrophage program [12]. DLL4 also links the vascular and stromal compartments: a recurrent capillary–fibroblast neighborhood co-expressed endothelial *DLL4* and fibroblast *NOTCH3*, and NOTCH blockade reduced the fibroblast matrix program [13]. A distinct *CXCL12*-expressing endothelial population impaired the differentiation of naive CD8^+^ T cells into cytotoxic effectors and recruited myeloid-derived suppressor cells (MDSCs) [14]. Analyses of a single onco-fetal single-cell resource associated PLVAP^+^ endothelial cells, FOLR2^+^ macrophages, and POSTN^+^ CAFs with an onco-fetal neighborhood and with postoperative relapse [12,15]. Related endothelial and macrophage programs also arise during liver injury and regeneration, so onco-fetal describes a shared transcriptional program rather than a tumor-specific lineage [12]. Separately, exploratory analyses linked related signatures to response in atezolizumab-bevacizumab-treated cohorts [16].

### 2.3. Myeloid Cells and Tumor-Associated Neutrophils

The hepatic myeloid compartment includes resident Kupffer cells and recruited monocyte-derived macrophages, which diversify into functionally and spatially distinct tumor-associated states [17]. Single-cell profiling of non-malignant human liver established transcriptionally distinct macrophage populations at baseline [18]. In HCC, imaging mass cytometry with supporting experimental analyses identified neighborhoods with opposing macrophage programs. Resident Kupffer cells high in programmed cell death ligand 1 (PD-L1) lay adjacent to CD8^+^ T cells high in programmed cell death protein 1 (PD-1), and this neighborhood was associated with worse outcome, whereas infiltrating macrophages expressing CD80, CD86, and HLA-DR occupied a separate neighborhood [19]. Recurrent marker-defined tumor-associated macrophage (TAM) states add another layer. FOLR2^+^ macrophages co-localized with PLVAP^+^ endothelial cells and regulatory T cells (Tregs) in analyses derived from the same onco-fetal dataset [12,15]. In a separate eight-patient resource, macrophages expressing *SPP1* accumulated with CAFs near the tumor boundary [20]. Together, these findings indicate that TAM function varies with cellular origin, activation state, and spatial position. Spatial proximity establishes an association, whereas causal interaction requires complementary experimental evidence.

Neutrophils form a distinct myeloid axis. In murine MASH-associated HCC, tumors resisted PD-1 blockade, whereas adding a CXCR2 antagonist altered the intratumoral neutrophil phenotype and improved tumor control [21]. Human evidence in the same study was transcriptomic and associative, distinguishing MASH-associated HCC from viral and alcohol-related HCC [21]. Separately, a *TREM1*-expressing neutrophil state in human HCC was associated with an immunosuppressive microenvironment and with immunotherapy response [22]. Neutrophil-directed treatment therefore rests on preclinical causality supported by human association, and its clinical evaluation in liver cancer remains ahead.

### 2.4. Conventional Lymphocytes and Tertiary Lymphoid Structures

The lymphoid compartment contains heterogeneous effector, dysfunctional, and regulatory states. Single-cell studies identify tumor-enriched PD-1-high CD8^+^ T-cell states and expanded Tregs [17]. The underlying liver disease also shapes lymphocyte function. In metabolic dysfunction-associated steatohepatitis, IL-15-mediated FOXO1 downregulation generates auto-aggressive CXCR6^+^PD-1^+^ CD8^+^ T cells that kill hepatocytes through P2X7 signaling independently of major histocompatibility complex (MHC) class I; these cells drive tissue injury rather than tumor-directed immunity [23]. Receptor expression alone can reflect inhibitory signaling or chronic stimulation. Exhaustion is therefore reserved here for antigen-specific states with demonstrated functional impairment, as discussed in Section 4.2.

B cells and tertiary lymphoid structures (TLS) illustrate why immune organization requires more than a measure of abundance. A high density of intratumoral B-cell-rich TLS after neoadjuvant immunotherapy was associated with pathologic response and longer relapse-free survival in HCC [24]. In a mouse model of iCCA, loss of *RORc*-expressing cells increased TLS number, and the resulting structures were enriched in CD8^+^ T cells, germinal-center B cells, and plasma cells and restrained tumor growth in a B-cell-dependent manner [25]. In resected human cholangiocarcinoma, TLS-positive tumors contained tumor-infiltrating lymphocytes carrying an exhaustion signature with PD-1 and LAG-3 expression [26]. TLS function therefore depends on location, cellular composition, and disease context as well as on number.

### 2.5. Antigen-Presenting, Innate, and Unconventional Lymphocytes

Evidence for several less frequently profiled populations is thinner than that for macrophages and conventional T cells. Dendritic-cell availability appears to be one control point. In mouse HCC, β-catenin activation reduced *Ccl5* expression and recruitment of conventional type 1 dendritic cells, whereas *Ccl5* re-expression restored immune surveillance [27]. In murine iCCA, CD40 agonism activated macrophages and dendritic cells and enhanced the response to PD-1 blockade [28]. Both findings are preclinical. In a single study, NK-cell infiltration tracked with an *SGMS2*-expressing macrophage population and with PD-1 treatment efficacy in HCC [29]. MAIT cells were less abundant in tumor than in adjacent liver and acquired a dysfunctional phenotype at the invasive margin, where spatial and functional experiments implicated nearby tumor-associated macrophages; the patient cohort was modest and drawn from one center [30]. HCC-infiltrating γδ T cells showed impaired proliferation, cytokine production, and cytotoxicity in a small patient series [31]. γδ T-cell infiltration has also been reported as a candidate correlate of response in an iCCA chemo-immunotherapy cohort (Section 5.2). NKT cells are discussed in Section 3.4, where microbial bile acid metabolism regulates their hepatic accumulation [32]. Across these populations most evidence derives from HCC, and iCCA-specific evidence remains confined to individual preclinical studies or cohorts; these gaps are identified where they occur rather than filled by extrapolation.

## 3. Structural, Metabolic, and Microbial Determinants

The cellular states described in Section 2 are shaped by local physical, metabolic, and microbial conditions. This section considers three interacting determinants: fibrosis-associated matrix remodeling, altered nutrient and metabolite availability, and gut-derived signals delivered through the portal circulation. These conditions differ across etiologies and tumor types. Selected pathways spanning distinct disease contexts, TME compartments, and levels of evidence are summarized in Table 1.

### 3.1. Matrix Stiffness and Fibrotic Remodeling

Fibrosis is both a major risk context for liver cancer and an active physical determinant of the TME. Activated HSCs and CAFs deposit and remodel extracellular matrix (ECM), increasing tissue stiffness [10,38]. Atomic force microscopy of resected HCC specimens showed that stiffness varied within individual tumors [39]. In HCC cell culture and mouse models, greater matrix stiffness promoted stem-like features and reduced sorafenib-induced apoptosis through integrin–YAP signaling, whereas lower-stiffness conditions favored proliferation [39]. Matrix mechanics therefore shift tumor-cell state rather than exerting a uniform effect on growth. Fibrosis also reorganizes cellular neighborhoods: single-cell profiling of human cirrhosis identified a structured fibrotic niche of scar-associated TREM2^+^CD9^+^ macrophages, PLVAP^+^ and ACKR1^+^ endothelial cells, and collagen-producing mesenchymal cells linked by profibrogenic signaling [38].

Three properties of the stroma are often reported together but behave differently: how much matrix is present, how fibroblasts are activated, and how collagen is organized. In iCCA, the matrix is compositionally reorganized rather than simply expanded, with accumulation of fibrillar collagens I, III and XII and periostin alongside loss of elastic fibers, reticular fibers and basement-membrane components, and with tumor collagen becoming straightened and aligned [40]. Quantity and architecture can point in opposite directions. In resected small-duct iCCA, a more abundant but less cellular stroma accompanied less vascular invasion, better differentiation and longer disease-free survival, whereas a more highly reticulated collagen network accompanied shorter overall survival [41]. Position matters as much as amount. In HCC, a marginal fibrotic ring of aligned, basement-membrane-rich matrix produced by C7^+^PDGFRA^+^ fibroblasts was associated with better survival, whereas dense cross-linked intratumoral stroma produced by FAP^+^POSTN^+^ fibroblasts was associated with worse survival and restricted T-cell infiltration [42]. In experimental iCCA, myofibroblasts at the tumor border restrained local proliferation while promoting invasion, whereas myofibroblasts within the tumor mass promoted proliferation [43]. The collagen-deletion experiment described in Section 2.1 points the same way. Fibroblast abundance measured without reference to location or matrix architecture therefore does not indicate the direction of the stromal effect. Estimates from dissociated tissue are also conservative, because fibroblasts are recovered inefficiently [11].

### 3.2. Metabolic Competition and Chronic-Liver Context

Tumor and immune cells compete for the same nutrients, and the chronically injured liver alters what is available. In murine non-hepatic tumors, intratumoral Tregs used monocarboxylate transporter 1 (MCT1)-mediated lactate uptake to sustain proliferation and suppressive function, and Treg-specific MCT1 deletion slowed tumor growth; this mechanism has not yet been demonstrated in liver tumors [33]. Lipid excess acts differently. In murine steatotic liver disease, linoleic acid induced mitochondrial reactive oxygen species and selective loss of intrahepatic CD4^+^ T cells, accelerating hepatocarcinogenesis, and human biopsies in the same study showed fewer intrahepatic CD4^+^ T cells in steatohepatitis than in viral hepatitis [34]. The metabolic environment of the tumor is thus partly set by the disease that preceded it, which is one reason etiology recurs as a variable in Section 4.5 and Section 6.

### 3.3. Tumor- and Stroma-Derived Metabolic Programs

Metabolic programs also originate within the stroma. Nicotinamide N-methyltransferase (NNMT) consumes S-adenosyl-methionine and lowers cellular methylation potential. It was identified as a master metabolic regulator of CAFs in ovarian cancer, where CAF NNMT expression reprogrammed the stroma and supported tumor progression [44]. Direct liver evidence has since emerged. In HCC, CAF NNMT binds EZH2 and impedes its nuclear translocation, reducing H3K27me3 at the *ANGPTL4* promoter and increasing ANGPTL4 secretion. ANGPTL4 promotes angiogenesis and engages GLUT1 on HCC cells, activating aerobic glycolysis, raising histone H3K18 lactylation, and upregulating PD-L1; in patient-derived xenografts and fibroblast-specific *Nnmt*-knockout mice, disrupting this axis restored CD8^+^ T-cell activity and synergized with anti-PD-L1 therapy [35]. On the malignant-cell side, activated HSCs induce NNMT in HCC cells, where it alters methylation potential and H3K27 methylation and stabilizes the CD44v3 isoform to promote invasion and metastasis [45]. Elevated intratumoral NNMT expression has also been associated with advanced disease and shorter disease-free survival [46].

Potent and selective NNMT inhibitors have been developed [47,48,49,50]. Pharmacologic evidence that CAF-directed NNMT inhibition restores antitumor immunity and improves immune-checkpoint blockade activity comes from ovarian cancer [51]. The liver evidence summarized above rests on genetic disruption in preclinical models, and no NNMT inhibitor has been evaluated clinically in liver cancer.

Oncometabolites provide a second, genotype-defined example, and in liver cancer they are largely an iCCA problem. Cancer-associated isocitrate dehydrogenase 1/2 (*IDH1/2*) mutations confer neomorphic enzymatic activity that generates D-2-hydroxyglutarate (D-2-HG) [52,53]. In experimental biliary cancer, D-2-HG blocked HNF-4α-dependent hepatocyte differentiation, and in cholangiocarcinoma models harboring mutant *IDH1*, it suppressed CD8^+^ T-cell activity and interferon-γ-responsive programs [36,53]. The therapeutic consequences are considered in Section 5.5.

### 3.4. The Gut–Liver Axis

The portal vein continuously exposes the liver to gut-derived nutrients, microbial products, and metabolites [54]. In chronic liver disease, intestinal barrier dysfunction and dysbiosis increase hepatic exposure to microbe-associated molecular patterns such as lipopolysaccharide, amplifying innate inflammatory and fibrogenic signaling [54]. Specific microbial metabolites act on defined hepatic compartments. In mouse models of primary and metastatic liver tumors, microbial conversion of primary to secondary bile acids lowered LSEC expression of CXCL16 and reduced accumulation of antitumor CXCR6^+^ NKT cells, and human nontumor liver showed a corresponding association between bile acids and CXCL16 expression [32].

In carcinogen-exposed obese mice, deoxycholic acid induced HSC senescence and a senescence-associated secretory phenotype that promoted HCC [37]. Gut microbiome composition has been associated with anti-PD-1 response in a small cohort of patients with HCC [55]. Most of this evidence is murine, and the human observations are correlative. The gut–liver axis is therefore best regarded at present as a source of mechanistic and biomarker hypotheses rather than a basis for patient selection.

## 4. Mechanisms of Immune Evasion and TME Heterogeneity

**Figure 2:**
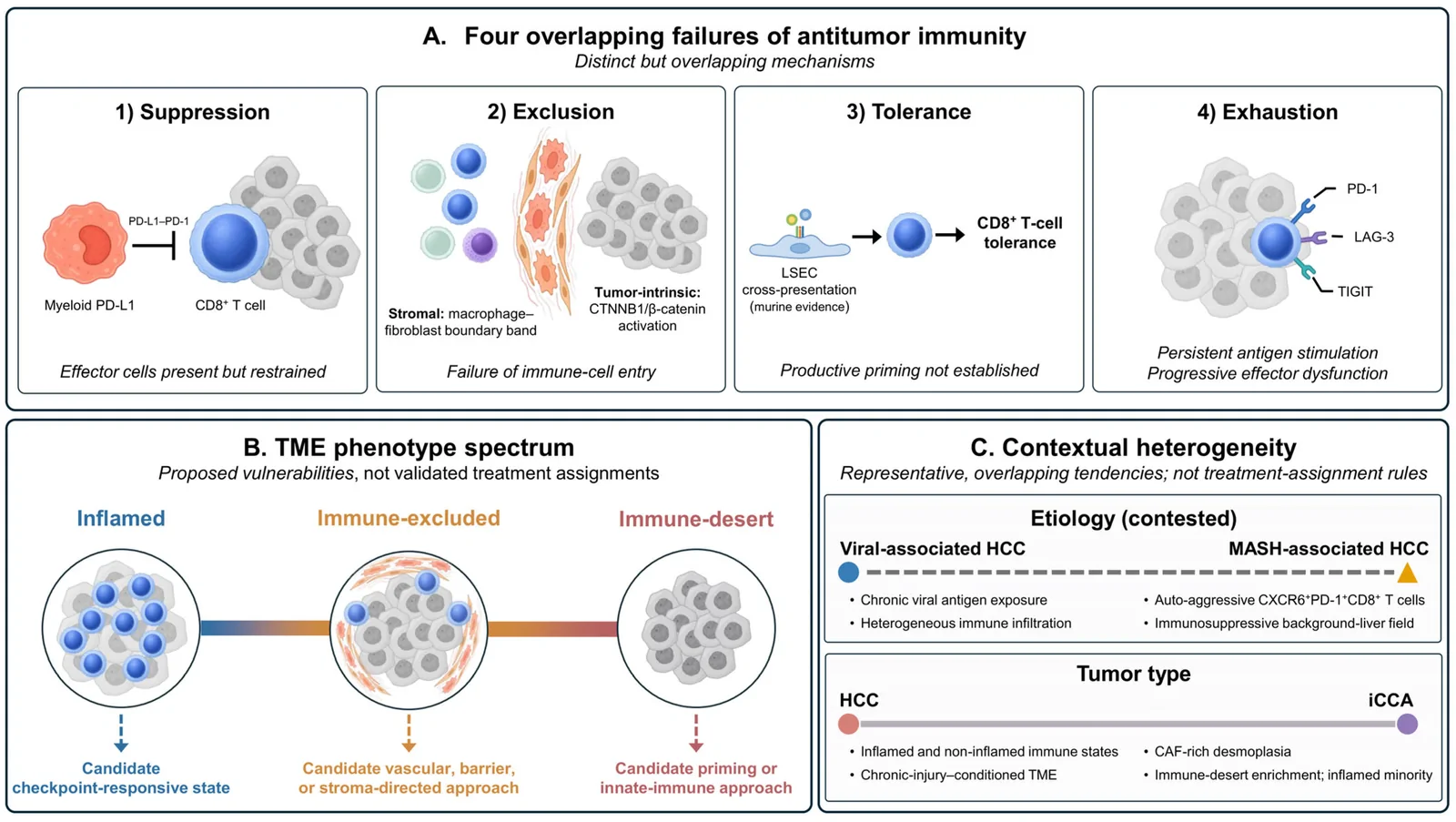
Mechanisms of immune evasion, phenotypes, and heterogeneity in liver cancer. (**A**) Four failures of antitumor immunity—suppression, exclusion, tolerance, and exhaustion—which overlap within individual tumors. (**B**) Inflamed, immune-excluded, and immune-desert patterns shown as an overlapping spectrum defined by the density and location of CD8^+^ T cells, each linked to a proposed therapeutic direction. (**C**) Overlapping tendencies reported by etiology and tumor type; disagreement among studies is discussed in Section 4.5. Cell types are distinguished by shape and label as well as by color; interaction symbols follow Figure 1. Exhaustion here means persistent, antigen-driven functional impairment, not marker expression alone.

The cellular states of Section 2 and the structural, metabolic, and etiologic conditions of Section 3 converge on several distinct failures of antitumor immunity. Immune suppression reduces the activity of effector cells already present; immune exclusion keeps lymphocytes away from malignant cells; tolerance limits productive priming to antigens encountered in the liver; and exhaustion is a persistent, antigen-driven dysfunctional T-cell state. These forms overlap within individual tumors but describe different problems (Figure 2). This section considers each in turn, then the tumor-intrinsic programs that produce them and the heterogeneity that determines which pattern a given tumor shows.

### 4.1. The Onco-Fetal Immunosuppressive Niche

The onco-fetal endothelial–myeloid niche illustrates how coordinated cellular reprogramming can sustain immune suppression in HCC. Ligand–receptor analysis predicts enriched immunoregulatory interactions between FOLR2^+^ TAMs and Tregs within this niche, and tumor tissue is relatively depleted of cytotoxic CD8^+^ T and NKT cells compared with adjacent liver [12]. VEGF and endothelial NOTCH signaling help maintain the PLVAP^+^ endothelial and fetal-like macrophage states, linking vascular reprogramming to local myeloid and regulatory programs [12]. In exploratory analyses of the same onco-fetal resource, the presence of these neighborhoods was associated with postoperative relapse and with signatures linked to response in atezolizumab–bevacizumab cohorts [12,15]. These are spatial and correlative observations, and they do not establish that the niche itself causes lymphocyte exclusion.

### 4.2. Checkpoint Suppression, Antigen Presentation, and T-Cell Dysfunction

Two liver-relevant failures can limit cytotoxic T-cell responses: inhibitory signaling within and around the tumor, and tolerogenic antigen presentation before effector function is established. In human HCC, Kupffer cells and peritumoral monocytes express PD-L1 (also known as B7-H1), and blocking the PD-L1–PD-1 interaction with tumor-infiltrating CD8^+^ T cells restores effector function [56,57]. Non-parenchymal myeloid cells are therefore functional sources of PD-L1-mediated suppression in the liver. Tumor-infiltrating T cells also express PD-1, LAG-3, TIGIT, and related receptors [17]. Such expression indicates inhibitory input or chronic stimulation rather than exhaustion, a term better reserved for antigen-specific states with demonstrated functional impairment [58]. Dysfunction within the effector compartment is itself heterogeneous. In iCCA, a study-defined precursor-exhausted CXCL13^+^CD39^+^CD103^+^ CD8^+^ T-cell state was associated with longer survival, whereas a terminally exhausted state was associated with shorter survival in the same published datasets [59]. In 140 patients with iCCA, a tissue-resident-memory-like CD39^+^PD-1^+^ CD8^+^ subset that lacked TIM-3 and LAG-3 expression was associated with shorter overall and recurrence-free survival in multivariable analysis [60]. In HCC sampled before checkpoint inhibitor therapy, a high proportion of exhausted CD8^+^ T cells within otherwise T-cell-enriched tumors was associated with shorter progression-free survival [61]. Senescence is a further route to effector failure: in mouse liver cancer, fibroblast-derived LAMA4 signaled through ITGA6 to induce CD8^+^ T-cell senescence, and silencing *LAMA4* restored function and synergized with anti-PD-1 [62].

A distinct failure can occur earlier, during antigen presentation. LSECs can cross-present antigen with limited co-stimulation and induce antigen-specific CD8^+^ T-cell tolerance, as demonstrated for tumor-derived antigens in mice [63]. Priming failure and effector failure call for different interventions, and current evidence addresses the second far better than the first.

### 4.3. Structural Immune Exclusion

Immune exclusion in HCC is defined by where lymphocytes sit rather than by how many are present. Imaging mass cytometry of 101 patients with HCC resolved three immune neighborhoods and three spatial immunotypes, separated by CD8^+^ T-cell density and by the ratio of parenchymal to stromal CD8^+^ T cells [61]. Stromal CD8^+^ T-cell density was similar in enriched and compartmentalized tumors, so entry into the parenchyma rather than recruitment to the tumor distinguished them. Among the patients treated with checkpoint inhibition, median progression-free survival was 8.3, 6.6, and 4.1 months across the enriched, compartmentalized, and depleted immunotypes, respectively [61]. An eight-patient integrated single-cell, spatial-transcriptomic, and imaging resource identified a boundary band of macrophages and fibroblasts separating lymphocyte-rich stroma from malignant tissue [9,20]. In mouse HCC, SPP1 blockade or macrophage-specific *Spp1* deletion increased intratumoral cytotoxic T cells and sensitized tumors to PD-1 blockade [20]. Reanalysis of the same sections assigned the boundary macrophages a different identity [64]. The architecture is therefore better established than the cell states that define it.

### 4.4. Tumor-Intrinsic Programs

Tumor-cell-intrinsic programs can produce these patterns. In mouse HCC, activated β-catenin signaling reduced chemokine-dependent dendritic-cell recruitment, limited CD8^+^ T-cell infiltration, and conferred resistance to PD-1 blockade [27]. In human HCC, Wnt–β-catenin activation was found in 49 tumors, of which 34 (69%) fell in non-inflamed classes and the remainder in the inflamed class; mutations outside the excluded class produced weak pathway activation or arose in tumors with high interferon signaling [65]. The phenotype is modifiable. Matrix metallopeptidase 9 (MMP9) mediated the immune effects of gain-of-function *CTNNB1* in mouse HCC, and MMP9 blockade restored CD8^+^ T-cell infiltration and cytotoxicity and improved anti-PD-1 efficacy [66]. Etiology modifies the same genotype: in 100 sequenced HCCs arising in steatotic liver disease, *CTNNB1* mutation was the dominant driver and acted through TNFRSF19 to repress senescence-associated cytokines, an effect reversed by a Wnt modulator in a syngeneic model [67]. Other oncogenic programs carry defined but thinner immune links. In *MYC*-driven HCC models, *MYC* overexpression suppressed innate immunity and MHC class I antigen presentation. Combined PD-L1 and cytotoxic T-lymphocyte-associated antigen 4 (CTLA-4) blockade, but neither agent alone, recruited proinflammatory antigen-presenting macrophages and restored antitumor activity [68]. In human HCC tissue microarrays, the frequency of tumor cells expressing transforming growth factor-β (TGF-β) correlated with the frequencies of FAP^+^ and α-SMA^+^ fibroblasts; fibroblast activation was not experimentally tested [69].

In iCCA, mutant IDH provides a defined metabolic–immune link, as discussed in Section 3.3, but most other genotype–microenvironment associations remain descriptive. Mutational profiles segregate with duct type: *IDH1* and *IDH2* mutations, *FGFR2* fusions, and *BAP1* alterations occur in small-duct tumors, whereas *KRAS*, *TP53*, and *SMAD4* alterations occur in large-duct tumors [70]. Spatial and immunohistochemical analysis found higher levels of MARCO^+^ macrophages and CTSE^+^ tumor cells in *KRAS*- and *TP53*-mutant tumors, and higher CTSE levels in *IDH1*- and *IDH2*-wild-type tumors [71]. In 25 patients treated with gemcitabine, cisplatin, and durvalumab, *TP53* mutation was the only independent predictor of shorter progression-free survival, and spatial profiling of selected cases showed sparse CD8^+^ T-cell infiltration and lower *TAP1* and *TAP2* expression in *TP53*-mutant tumors; this cohort was small and retrospective [72]. Mechanistic evidence linking *FGFR2* fusions, *KRAS*, or *BAP1* alterations to microenvironmental organization remains limited, and the reported associations are therefore hypothesis-generating.

### 4.5. Heterogeneity by Etiology, Spatial State, and Tumor Type

HCC separates reproducibly into inflamed and non-inflamed tumors. A revised immunogenomic classification from the group that described the original immune class defined an inflamed class comprising 37% of tumors, an intermediate class enriched for *TP53* mutation, and an excluded class enriched for *CTNNB1* mutation [73]. A 20-gene signature captured about 90% of inflamed tumors and was overexpressed in responders within an external cohort treated with checkpoint inhibitors [65]. The classification was derived in 240 patients and validated in a further 660 [65]. The non-inflamed group therefore spans intermediate and excluded patterns rather than a single immune-desert state.

Etiology adds a further layer, and its spatial consequences are contested. MASH-associated HCC arises against an immunosuppressive background liver and carries distinct mutational and transcriptomic features [74]. In steatohepatitis models, PD-1 blockade expanded a CXCR6^+^PD-1^+^ CD8^+^ T-cell population with the tissue-damaging features described in Section 2, without restoring tumor surveillance [23,75]. Post hoc clinical analyses have suggested less benefit from checkpoint blockade in non-viral HCC, although mixed subgroups and observational designs limit what can be concluded [75]. Imaging mass cytometry of 16 MASH-associated and 11 virus-associated HCCs found an immune-cell gradient that was densest in adjacent tissue and declined toward the tumor in MASH-HCC only [76]. A larger spatial series of 101 patients found no clear association between underlying liver disease and spatial immunotype [61]. Etiology therefore shifts the probability of particular immune states without determining them.

In iCCA, the immune description is incomplete without duct type. S100P and SPP1 were mutually exclusive in malignant cells and separated perihilar large-duct from peripheral small-duct tumors in 92.5% of cases, with validation in a 201-patient tissue microarray [77]. Large-duct tumors carried worse survival, more PD-1^+^CD8^+^ T cells, fewer CD3^+^ T and CD56^+^ NK cells, and CCL18^+^ macrophages [77]. A transcriptomic classification defined four microenvironment-based subtypes, including an immune-desert group of roughly 45% and an inflamed minority of about 11% with better survival [78]. Imaging of 155 patients with validation in 214 identified high-risk tumors that were inflamed yet prognostically adverse [79], and in 139 resected iCCAs, CD4^+^ rather than CD8^+^ density carried the survival signal [80]. Neither iCCA nor HCC should therefore be assigned a uniform immune phenotype based on tumor type alone [6].

## 5. Therapeutic Implications

The mechanisms in Section 2, Section 3 and Section 4 identify specific microenvironmental barriers that may be therapeutically modified. Therapeutic development has moved faster than microenvironment-based patient selection: several regimens now have established survival benefit, yet no microenvironmental feature selects treatment for an individual patient. This section is organized by the barrier each strategy is intended to modify—aberrant vasculature, checkpoint suppression, myeloid and stromal sequestration, metabolic restriction, and deficient effector function—and keeps demonstrated efficacy separate from evidence that the proposed mechanism mediates it. HCC supplies most of the clinical evidence, and iCCA serves as a biologically distinct comparator. Table 2 summarizes trial designs and results. Clinical evidence is current through August 2026.

### 5.1. Vascular–Immune and Dual-Checkpoint Blockade in HCC

VEGF drives disorganized angiogenesis and immunosuppressive signaling, providing a rationale for combined vascular and immune blockade. In mouse HCC, combined VEGF receptor 2 and PD-1 blockade increased pericyte-covered vessels and CD8^+^ T-cell infiltration while reducing hypoxia, Tregs, and CCR2^+^ monocytes [105]. In systemic treatment-naive unresectable HCC, atezolizumab plus bevacizumab improved overall and progression-free survival over sorafenib in IMbrave150 and was established as a first-line option [81]. Efficacy is established, but the trial measured no microenvironmental endpoint. Human tissue evidence is small: in seven specimens resected after neoadjuvant cabozantinib plus nivolumab, pathologic response tracked with immune-rich regions high in antigen processing and presentation [106], and in 54 anti-PD-1-treated patients at one center an FMO2^+^ fibroblast state was associated with response [107].

Dual checkpoint blockade targets complementary inhibitory steps: CTLA-4 blockade can augment T-cell priming, whereas PD-1 or PD-L1 blockade acts principally on previously activated intratumoral T cells [98]. A single priming dose of tremelimumab added to durvalumab—the STRIDE (Single Tremelimumab Regular Interval Durvalumab) regimen—improved overall survival over sorafenib in HIMALAYA [83], and nivolumab plus ipilimumab improved overall survival over investigator-selected lenvatinib or sorafenib in CheckMate 9DW [84]. Both are established first-line options. In CheckMate 9DW, mortality was higher with nivolumab plus ipilimumab during the first six months before the curves separated; deaths judged unrelated to treatment contributed substantially, and most treatment-related deaths were hepatic [84]. This early hazard is the main practical constraint. For neither regimen has the benefit been traced to a defined microenvironmental interaction in patients.

### 5.2. Chemo-Immunotherapy in Advanced Biliary Tract Cancer

iCCA is treated within the biliary tract cancer group, in which two randomized trials changed first-line therapy. In the primary TOPAZ-1 analysis, durvalumab added to gemcitabine and cisplatin improved overall survival in 685 patients; hazard ratio 0.80 (95% confidence interval 0.66–0.97, *p* = 0.021) [85]. At a median follow-up of 41.3 months the hazard ratio was 0.74 (0.63–0.87), with 36-month survival of 14.6% versus 6.9% [86]. In KEYNOTE-966, pembrolizumab added to the same backbone improved overall survival in 1069 patients, hazard ratio 0.83 (0.72–0.95, one-sided *p* = 0.0034), with median survival of 12.7 versus 10.9 months [87]. Successive analyses of one trial give different hazard ratios, so each estimate should be named with its analysis.

Intrahepatic tumors accounted for 56% of TOPAZ-1 and 59% of KEYNOTE-966, so application to iCCA rests on substantial representation, not remote extrapolation [85,87]. The treatment effects were nevertheless estimated across biliary tract cancers as a whole, and neither trial was powered to establish efficacy within an individual anatomical subgroup. The KEYNOTE-966 investigators note that the disproportionate enrolment of intrahepatic tumors may partly reflect the requirement for tumor tissue at entry, these tumors being more accessible to sampling [87]. Microenvironmental mediation is untested: in a single-arm, single-center conversion cohort of 66 patients with iCCA, post-resection specimens from the 10 patients who reached surgery showed higher PD-L1 and CD8 and lower α-SMA than pretreatment biopsies, without paired sampling or a comparator [108].

### 5.3. Locoregional Therapy as Microenvironmental Rewiring

Locoregional therapies reduce tumor burden and can also alter the microenvironment, but these effects are modality specific. Ischemic, thermal, mechanical, and radiation injury differ in antigen preservation, vascular effects, hypoxia, and release of damage-associated molecular patterns; preclinical studies suggest they promote dendritic-cell activation and systemic T-cell priming to differing degrees [109]. In murine tumor models, including HCC, histotripsy released tumor antigens, generated CD8^+^ T-cell-dependent local and abscopal responses, and enhanced checkpoint-blockade activity [92]. Human evidence is confined to feasibility studies and case-level observations [93].

Transarterial chemoembolization (TACE) produces ischemic necrosis and post-embolization hypoxia that may release tumor antigens and danger signals while inducing VEGF [88,109]. Adding systemic therapy to TACE improved progression-free survival in two published phase 3 trials—durvalumab plus bevacizumab in EMERALD-1, in which durvalumab plus TACE was not superior to TACE alone, and lenvatinib plus pembrolizumab in LEAP-012 [89,90]. The phase III EMERALD-3 trial, presented as a conference abstract at the 2026 ASCO Annual Meeting, reported improved progression-free survival with STRIDE plus lenvatinib and TACE versus TACE alone [91]. Overall survival is not yet established for any of these regimens, and each added high-grade toxicity [88,89,91]. TACE-based combinations have therefore not become routine care, and the proposed immune mechanism has not been tested as a mediator.

### 5.4. Myeloid- and Stroma-Directed Strategies

Myeloid and fibroblast states contribute to immune suppression and exclusion (Section 2 and Section 4), but therapeutic evidence in liver cancer is largely preclinical. Myeloid-directed strategies intervene at several points: colony-stimulating factor 1 receptor (CSF1R) inhibition alters macrophage survival and state; CD40 agonism activates antigen-presenting programs; and CD47–signal regulatory protein α blockade removes an antiphagocytic signal [110]. In murine iCCA, CD40 agonism activated macrophages and dendritic cells and improved the response to anti-PD-1 [28]. Blocking myeloid recruitment or survival is less straightforward than first proposed: resident and recruited hepatic macrophages can have divergent functions (Section 2.3), and in cholangiocarcinoma models both genetic *Ccr2* deletion and CSF1R blockade left tumor burden unchanged, because CAF-derived CXCL2 recruited granulocytic myeloid-derived suppressor cells in compensation [95]. SPP1 blockade or macrophage-specific *Spp1* deletion improved anti-PD-1 activity in mouse HCC, the therapeutic counterpart of the boundary band in Section 4.3 [20]. Anti-TREM2 antibody enhanced anti-PD-1 efficacy in mouse sarcoma, colorectal, and mammary tumors, with no liver-cancer counterpart [94].

Stroma-directed approaches must be aimed at fibroblast subpopulations rather than at stroma as a whole. In genetically engineered mouse iCCA, hepatic stellate cells give rise to most CAFs, and depleting those CAFs by two independent strategies reduced tumor burden [10]. Deleting type I collagen in the same fibroblasts lowered matrix stiffness without changing tumor growth, which separates matrix amount from fibroblast activation as targets [10]. Broad fibroblast depletion has failed in other desmoplastic cancers and has sometimes accelerated progression [111]. Mediator-directed approaches have preclinical support: blocking placental growth factor shifted iCCA CAFs to a quiescent, low-collagen state, reopened collapsed vessels and improved gemcitabine–cisplatin efficacy in orthotopic mouse models [97]; focal adhesion kinase inhibition with anti-PD-1 improved control of orthotopic mouse HCC [96]. TGF-β and the CXCL12–CXCR4 axis have given inconsistent early-phase results with checkpoint blockade [98], and the stromal metabolic node in Section 3.3 is at the same preclinical stage.

### 5.5. Genotype- and Metabolism-Directed Strategies

iCCA provides a biologically distinct example in which a tumor-derived oncometabolite reshapes immunity. Mutant IDH1 generates D-2-HG, which suppressed CD8^+^ T-cell function and interferon-responsive programs in preclinical cholangiocarcinoma models; IDH1 inhibition restored these responses in mice [36]. In ClarIDHy, ivosidenib improved progression-free survival over placebo in previously treated, advanced *IDH1*-mutant cholangiocarcinoma [99]. The trial measured no immune endpoint, so the immune mechanism is supported only in models. Other metabolic interventions, including microbiome modulation and targeting of lactate, adenosine, arginine, glutamine, or lipid metabolism, remain preclinical.

### 5.6. Emerging Immunotherapies and Engineered Cells

Chimeric antigen receptor (CAR) cells and therapeutic vaccines show the widest gap between rationale and evidence. CAR T cells directed against glypican-3 have reached patients: in two phase I studies, 13 patients with advanced HCC were treated, two had confirmed partial responses, overall survival was 42.0% at one year and 10.5% at three years, cytokine release syndrome was grade 1 or 2 and reversible in eight patients, and one patient died of grade 5 cytokine release syndrome [100]. That is early clinical activity at a measurable cost. Other engineered platforms remain preclinical: a CAR macrophage directed against NKG2D ligands controlled tumor growth in mice [101], glypican-3-directed CAR natural killer cells derived from the NK-92 line were active against glypican-3-positive HCC xenografts [102], and B7-H3-directed CAR T cells produced durable responses in orthotopic and patient-derived xenograft models of iCCA [103]. Engineered cellular therapy is therefore early clinical in HCC and preclinical in iCCA.

HepaVac-101 evaluated therapeutic vaccination principally through immunogenicity endpoints. In this single-arm phase I/II study, 82 patients were screened and 22 were vaccinated; the primary endpoints were safety, tolerability, and antigen-specific T-cell response, and vaccine-induced responses were detected in 37% of patients for class I and 53% for class II antigens [104]. The design supports a claim of immunogenicity, not of efficacy.

Checkpoint receptors beyond PD-1 and CTLA-4 are earlier still, and iCCA shows why target presence must be separated from therapeutic activity. In 50 resected iCCA specimens, TIM-3 on CD4^+^ cells was denser within the tumor than at the tumor–liver interface, and no therapeutic intervention was tested [112]. In a different iCCA cohort, the tumor-restricted CD8^+^ subset associated with worse outcome expressed neither TIM-3 nor LAG-3 [60]. These studies establish that TIM-3 is present in part of the iCCA immune compartment, but neither identifies the population that would be targeted, and no TIM-3 inhibitor has shown activity in this disease. CTLA-4 blockade remains the checkpoint strategy beyond PD-1 with established efficacy in HCC (Section 5.1).

### 5.7. Natural Products and Traditional Medicine

Compounds used in traditional Chinese medicine are widely given alongside standard treatment for liver cancer in some regions, and several herbal-derived compounds modify the microenvironment in preclinical models. Ginsenoside Rh1 is the clearest worked example. In mouse HCC it suppressed the glucocorticoid receptor, raised MHC class I expression on tumor cells, promoted dendritic-cell maturation and CD8^+^ T-cell activation, and increased the antitumor effect of lenvatinib; it slowed tumor growth in immunocompetent mice but not in nude mice, so the effect required host adaptive immunity [113]. The model is a subcutaneous mouse tumor.

Human evidence is weaker and is not linked to that mechanism. A meta-analysis of 19 randomized trials and 1448 patients reported that ginsenosides added to transarterial chemoembolization, surgery, or chemotherapy improved disease control, one- and two-year survival, and quality of life, while stating that the pooled trials were generally of low quality [114]. Network pharmacology and docking studies do not provide stronger evidence. Natural products are best treated here as a preclinical source of microenvironment-directed mechanisms rather than as an established adjunct.

## 6. Translational Challenges and Biomarker Readiness

The principal obstacle to translating this biology is not a shortage of plausible targets but heterogeneity, and the shape of that heterogeneity is better defined than it was. The liver TME varies by tumor type, etiology, disease stage, spatial organization, and prior treatment, and these dimensions overlap rather than define stable categories. Multiregional single-cell profiling of seven liver cancers found that malignant cells clustered by patient rather than by region [115]. Inferred tumor–macrophage communication networks were also largely stable across separate regions of one tumor, though the authors report lower regional stability in iCCA than in HCC [115]. Between-patient differences therefore exceeded within-tumor differences for these features. A single biopsy can still miss spatially restricted features: boundary architecture, invasive-front states, and treatment-induced change. This limitation applies at that resolution, not to biopsies in general.

Clinical efficacy and biomarker readiness should be judged separately. Anti-VEGF plus immune checkpoint inhibition and dual checkpoint blockade have established efficacy in advanced HCC, yet regimen choice still rests on conventional clinical factors, and no microenvironmental feature currently selects between them [116]. Inflamed, immune-excluded, and immune-desert phenotypes are useful organizing frameworks; the phenotype-to-therapy links shown in Figure 2 are proposed vulnerabilities rather than validated treatment assignments. Onco-fetal signatures, the macrophage–fibroblast boundary band, POSTN^+^ CAF states, TREM2^+^ macrophage states, and microbiome signatures are candidate biomarkers, each associated with response, prognosis, or pharmacodynamic change. Predictive utility requires a further step: evidence of differential benefit from one treatment relative to an appropriate comparator [116].

Assay readiness is a separate problem from biology, and the spatial literature is at present not comparable across studies. The word neighborhood denotes a cell and its neighbors within 4 micrometers in one HCC imaging study, the forty nearest cells within 75 micrometers in another, and direct algorithmic adjacency in a third [19,61,76]. The second of these tests cell–cell interaction on a separate 15-micrometer expansion, and a later iCCA study adopts the same 75-micrometer definition [61,79]. Thresholds are often derived and refined within the same data. In the 75-micrometer study, the parenchymal-to-stromal ratio cut-off came from the discovery distribution and was then revised using the discovery and treated cohorts together, so the improved separation is internal rather than independent [61]. Morphometry shows a related limitation: an intratumoral homogeneity analysis in resected iCCA identified collagen reticulation as the only stromal parameter stable enough to assess on a biopsy [41]. Dissociation-based methods add a further bias by under-recovering fibroblasts (Section 3.1). Harmonized definitions and prespecified cut-offs are prerequisites for comparing these studies, let alone for using them clinically.

Etiology may modify immune ecology while falling short of a stand-alone treatment rule. Experimental MASH-associated HCC models show that PD-1 blockade can expand tissue-damaging CD8^+^ T-cell programs [23,75]; the contested clinical counterpart is set out in Section 4.5. Etiology should therefore inform prospective, prespecified analyses rather than be used on its own to withhold checkpoint blockade.

Locoregional therapy remodels the microenvironment as well as reducing tumor burden, but antigen release and immune activation are steps in a mechanism rather than evidence of survival benefit or of an optimal sequence. Positive results from TACE-based trials apply to specific regimens and populations; timing, sequence, duration, and preservation of liver function are still open [88]. Single-cell and spatial profiling, circulating tumor DNA, and microbiome analysis remain research platforms rather than treatment-selection assays, limited by sampling, standardization, reproducibility, cost, and turnaround time. Before any classifier guides treatment, prospective studies with predefined cut-offs and external validation should show that differential benefit rather than prognosis alone.

## 7. Conclusions

Liver tumors do not create their microenvironment de novo. Hepatic malignancies reuse hepatic programs that normally maintain immune tolerance, tissue repair, vascular adaptation, stromal remodeling, and metabolic homeostasis. As these programs are redirected by malignant cells and by chronic liver injury, interactions among tumor, immune, stromal, vascular, and metabolic compartments come to sustain tumor growth and immune escape. The liver TME is therefore better understood as an integrated, evolving ecosystem than as a catalog of cells or pathways.

Read as a comparison, HCC and iCCA converge on a shared core and diverge around it. Both are organized around myeloid and stromal states rather than around lymphocyte number alone. Checkpoint blockade has entered first-line therapy for HCC and, within the broader biliary tract cancer group, for iCCA. What surrounds that core differs: desmoplastic stroma and fibroblast heterogeneity carry far more weight in iCCA, duct type has no counterpart in HCC, and a tumor-derived oncometabolite provides a genotype-defined metabolic–immune route that HCC does not offer. The evidence bases also diverge. HCC supplies most of the mechanistic and clinical data, whereas iCCA evidence often rests on single cohorts or on re-analyses of shared datasets, and this review states where that is so, rather than filling the gap by extrapolation.

Patient selection has not kept pace with therapeutic development. Anti-VEGF plus checkpoint inhibition and dual checkpoint blockade in HCC, and chemo-immunotherapy in biliary tract cancer, are established on survival endpoints. Yet none of these trials tested the microenvironmental mechanism proposed to explain them, and no phenotype, spatial or single-cell signature, circulating tumor DNA measure, microbiome feature, or multi-omics classifier yet assigns treatment prospectively. Closing that gap calls for a different kind of study rather than more descriptive profiling: harmonized spatial definitions, cut-offs fixed before the outcome is examined, and trials designed to test differential benefit. Organizing interventions by the barrier they are intended to modify clarifies the gap by separating what has been shown to work from what has been shown to explain why. On present evidence, the liver TME is a biologically coherent framework that informs treatment selection without yet determining it.

## Figures and Tables

**Table 1:** Selected metabolic and microbial pathways with immune or stromal consequences relevant to liver cancer.

Pathway	Cancer Type	TME Compartment	Model or Population	Mechanism or Consequence	Evidence Maturity	Ref.
Lactate uptake through MCT1	Non-hepatic tumors	Lymphoid (regulatory T cells)	Murine non-hepatic tumors; Treg-specific MCT1 deletion	MCT1-mediated lactate uptake sustains regulatory T-cell proliferation and suppressive function; Treg-specific deletion slowed tumor growth	Preclinical; not demonstrated in liver tumors	[33]
Linoleic acid accumulation	HCC in steatotic liver disease	Lymphoid (CD4^+^ T cells)	Murine steatotic liver disease; human liver biopsies in the same study	Mitochondrial reactive oxygen species and selective loss of intrahepatic CD4^+^ T cells, accelerating hepatocarcinogenesis; fewer intrahepatic CD4^+^ T cells in human steatohepatitis than in viral hepatitis	Preclinical, with an associated human observation	[34]
NNMT–ANGPTL4 axis	HCC	Stromal (CAFs), acting on tumor cells and vasculature	Human HCC tissue; patient-derived xenografts; fibroblast-specific Nnmt-knockout mice	CAF NNMT binds EZH2 and impedes its nuclear translocation, lowering H3K27me3 at the *ANGPTL4* promoter and increasing ANGPTL4 secretion; ANGPTL4 promotes angiogenesis and engages tumor-cell GLUT1, increasing aerobic glycolysis, H3K18 lactylation, and PD-L1 expression; axis disruption restored CD8^+^ T-cell activity and synergized with anti-PD-L1 therapy	Preclinical, by genetic disruption; no NNMT inhibitor evaluated clinically in liver cancer	[35]
Mutant *IDH1/2* and D-2-hydroxyglutarate	iCCA	Tumor cell, acting on lymphoid cells	Experimental biliary cancer; *IDH1*-mutant cholangiocarcinoma models	Neomorphic enzyme activity generates D-2-HG, which suppresses CD8^+^ T-cell activity and interferon-γ-responsive transcriptional programs	D-2-HG production established; the immune effect preclinical	[36]
Microbiota-derived secondary bile acids	HCC and liver metastases	Endothelial (LSECs), acting on lymphoid cells	Mouse models of primary and metastatic liver tumors; human nontumor liver	Lower LSEC CXCL16 expression and reduced hepatic accumulation of antitumor CXCR6^+^ NKT cells; bile acids and CXCL16 expression correlated in human liver	Preclinical, with an associated human correlation	[32]
Deoxycholic acid	HCC, obesity-associated	Stromal (hepatic stellate cells)	Carcinogen-exposed obese mice	Hepatic stellate cell senescence and a senescence-associated secretory phenotype that promotes HCC	Preclinical	[37]

Abbreviations: CD, cluster of differentiation; CXCL16, C-X-C motif chemokine ligand 16; D-2-HG, D-2-hydroxyglutarate; HCC, hepatocellular carcinoma; iCCA, intrahepatic cholangiocarcinoma; IDH1/2, isocitrate dehydrogenase 1/2; LSEC, liver sinusoidal endothelial cell; MCT1, monocarboxylate transporter 1; NKT, natural killer T cell; Treg, regulatory T cell. Note: Evidence maturity refers to the specific immune or stromal consequence described, not to the existence of the metabolite or pathway itself. Model context is stated explicitly where evidence was obtained outside liver cancer.

**Table 2:** Microenvironment-informed therapeutic strategies in liver cancer.

Strategy	Microenvironmental Target or Rationale	Key Evidence	Evidence Maturity
Anti-VEGF plus ICI	Counter VEGF-mediated immunosuppression and aberrant tumor vasculature	IMbrave150: OS HR 0.58; updated median OS 19.2 versus 13.4 months (HR 0.66) [81,82]	First-line efficacy established; vascular and myeloid mediation clinically unproven
Dual ICI	CTLA-4 blockade augments T-cell priming; PD-1/PD-L1 blockade acts on previously activated intratumoral T cells	HIMALAYA (STRIDE): OS HR 0.78. CheckMate 9DW: OS HR 0.79; HR 1.65 during months 0–6, 0.61 thereafter [83,84]	First-line efficacy established; early hazard specific to nivolumab plus ipilimumab
Chemo-immunotherapy in biliary tract cancer	Add PD-1/PD-L1 blockade to gemcitabine–cisplatin; microenvironmental mediation untested	TOPAZ-1: OS HR 0.80 (0.66–0.97) at primary analysis and 0.74 (0.63–0.87) at 41.3 months [85,86]. KEYNOTE-966: OS HR 0.83 (0.72–0.95) [87]. Intrahepatic tumors constituted 56% and 59% of the two trial populations	First-line efficacy established for biliary tract cancer as a whole; not powered to establish efficacy within an individual anatomical subgroup
TACE plus systemic therapy	Ischemic necrosis and hypoxia may release tumor antigens and damage-associated molecular patterns while inducing VEGF	EMERALD-1: PFS HR 0.77; durvalumab plus TACE not superior (HR 0.94). LEAP-012: PFS HR 0.66; follow-up OS not significant. Both increased high-grade toxicity. EMERALD-3 (2026 ASCO conference abstract): significantly improved PFS with STRIDE plus lenvatinib and TACE; a favorable descriptive result was observed with STRIDE plus TACE without lenvatinib; OS was not significant at an immature interim analysis [88,89,90,91]	Regimen-specific randomized PFS benefit; OS and routine-care role unresolved
Ablation or radiation plus ICI	Modality-dependent tumor-antigen and damage-associated molecular pattern release may promote local immune activation	Histotripsy produced CD8^+^ T-cell-dependent local and abscopal effects in mouse models, including HCC; evidence for thermal ablation and stereotactic radiation combinations remains preclinical or early clinical [92,93]	Preclinical or early clinical; systemic immune benefit and combination efficacy unproven
Myeloid-directed	Modify macrophage survival or state, monocyte recruitment, antigen presentation, phagocytosis, or the macrophage–fibroblast boundary band	SPP1 disruption enhanced anti-PD-1 activity in mouse HCC [20]. CD40 agonism improved the anti-PD-1 response in murine iCCA [28]. Anti-TREM2 antibody enhanced anti-PD-1 efficacy in mouse sarcoma, colorectal and mammary tumors, with no liver-cancer data [94]. Genetic *Ccr2* deletion and CSF1R blockade were each offset by compensatory granulocytic myeloid-derived suppressor cells [95]	Preclinical; no established efficacy in liver cancer
Stroma-directed	Reduce immune exclusion or suppressive stromal–immune signaling via fibroblast subpopulations or their mediators	Focal adhesion kinase inhibition with anti-PD-1 improved control of orthotopic mouse HCC [96]. Placental growth factor blockade reduced collagen and stiffness and improved chemotherapy efficacy in orthotopic mouse iCCA [97]. TGF-β and CXCL12–CXCR4 combination data are inconsistent [98]	Preclinical; broad fibroblast depletion has failed in other desmoplastic cancers
Metabolic: IDH1 inhibitor	Inhibit mutant IDH1 to reduce D-2-HG production and relieve oncometabolite-associated immune suppression	ClarIDHy: ivosidenib PFS HR 0.37 versus placebo in previously treated *IDH1*-mutant cholangiocarcinoma; no immune endpoint [36,99]	Efficacy established in selected *IDH1*-mutant cholangiocarcinoma; immune mechanism supported only in models
Engineered cells and vaccines	Redirect effector cells to a tumor antigen, or prime a response to one	CAR-glypican-3 T cells, HCC phase I: 13 patients, 2 partial responses, 3-year OS 10.5%, one fatal grade 5 cytokine release syndrome [100]. CAR macrophage and CAR natural killer cells: mouse and xenograft only [101,102]. B7-H3 CAR T in iCCA: xenograft only [103]. HepaVac-101 vaccine: 22 vaccinated, immunogenicity endpoints only [104]	Early clinical in HCC, preclinical in iCCA; no established efficacy

Abbreviations: CAR, chimeric antigen receptor; CTLA-4, cytotoxic T-lymphocyte-associated antigen 4; D-2-HG, D-2-hydroxyglutarate; HCC, hepatocellular carcinoma; HR, hazard ratio; ICI, immune checkpoint inhibitor; IDH1, isocitrate dehydrogenase 1; OS, overall survival; PD-1, programmed cell death protein 1; PD-L1, programmed cell death ligand 1; PFS, progression-free survival; SPP1, secreted phosphoprotein 1; STRIDE, Single Tremelimumab Regular Interval Durvalumab; TACE, transarterial chemoembolization; TGF-β, transforming growth factor-β; VEGF, vascular endothelial growth factor.

## Data Availability

No new data were created or analyzed in this study. Data sharing is not applicable to this article.

## Supplementary Materials

The following supporting information can be downloaded at: https://www.mdpi.com/article/10.3390/cancers18162696/s1, Table S1. Module 1 search: single-cell, spatial, and stromal architecture. All unique records were screened by title and abstract; records that could not be resolved at that stage were carried to full-text review rather than excluded. Searches were run on 10 August 2026 and covered records published from 1 January 2016 through the search date. Table S2. Modules 2 to 7: targeted searches for immune populations, tumor-intrinsic programs, treatment, and natural products. Records were ranked by relevance to the prespecified topics, and a defined number of the highest-ranked records from each module was screened by title. Searches were run on 11 August 2026.

## Abbreviations

The following abbreviations are used in this manuscript:

ACKR1	atypical chemokine receptor 1
ANGPTL4	angiopoietin-like 4
ASCO	American Society of Clinical Oncology
α-SMA	alpha smooth muscle actin
B7-H1	B7 homolog 1
CAF	cancer-associated fibroblast
CAR	chimeric antigen receptor
CCR2	C-C motif chemokine receptor 2
CD	cluster of differentiation
CSF1R	colony-stimulating factor 1 receptor
CTLA-4	cytotoxic T-lymphocyte-associated antigen 4
CTNNB1	catenin beta 1
CXCL12	C-X-C motif chemokine ligand 12
CXCL16	C-X-C motif chemokine ligand 16
CXCR4	C-X-C motif chemokine receptor 4
CXCR6	C-X-C motif chemokine receptor 6
D-2-HG	D-2-hydroxyglutarate
DLL4	delta-like canonical Notch ligand 4
ECM	extracellular matrix
EZH2	enhancer of zeste homolog 2
FAP	fibroblast activation protein
FOLR2	folate receptor beta
FOXO1	forkhead box O1
GLUT1	glucose transporter 1
H3K18	histone H3 lysine 18
H3K27me3	trimethylated histone H3 lysine 27
HCC	hepatocellular carcinoma
HGF	hepatocyte growth factor
HNF-4α	hepatocyte nuclear factor 4 alpha
HSC	hepatic stellate cell
iCCA	intrahepatic cholangiocarcinoma
IDH1/2	isocitrate dehydrogenase 1/2
IL-15	interleukin 15
LAG-3	lymphocyte activation gene 3
LSEC	liver sinusoidal endothelial cell
MAIT	mucosal-associated invariant T
MASH	metabolic dysfunction-associated steatohepatitis
MCT1	monocarboxylate transporter 1
MDSC	myeloid-derived suppressor cell
MET	MET proto-oncogene receptor tyrosine kinase
MHC	major histocompatibility complex
MMP9	matrix metallopeptidase 9
NK	natural killer
NKT	natural killer T
NNMT	nicotinamide N-methyltransferase
P2X7	P2X purinoceptor 7
PD-1	programmed cell death protein 1
PD-L1	programmed cell death ligand 1
PLVAP	plasmalemma vesicle-associated protein
POSTN	periostin
SPP1	secreted phosphoprotein 1
STRIDE	Single Tremelimumab Regular Interval Durvalumab
TACE	transarterial chemoembolization
TAM	tumor-associated macrophage
TGF-β	transforming growth factor beta
TIGIT	T-cell immunoreceptor with immunoglobulin and ITIM domains
TLS	tertiary lymphoid structure
TME	tumor microenvironment
Treg	regulatory T cell
TREM2	triggering receptor expressed on myeloid cells 2
VEGF	vascular endothelial growth factor
YAP	Yes-associated protein
