# Supplementary material for: The Liver Tumor Microenvironment in Hepatocellular Carcinoma: Comparisons with Intrahepatic Cholangiocarcinoma and Therapeutic Implications
Source: Cancers (Basel). 2026 Aug 20;18(16):2696. doi: 10.3390/cancers18162696 (PMC13510931; doi:10.3390/cancers18162696)
Supplement: Supplementary file 1 [file cancers-18-02696-s001.zip › cancers-4500759-supplementary.pdf]

Supplementary Methods

These searches were run to update an existing evidence base. References cited in the submitted version of the manuscript were reassessed against the same criteria for this revision and are not counted in the tables below. Reference lists of selected primary studies and recent reviews were examined selectively, and references nominated by the peer reviewers were assessed against the same criteria; these sources are likewise not counted below.

All searches were run in PubMed through the National Center for Biotechnology Information E-utilities interface and were restricted to English-language records. The Module 1 searches were run on 10 August 2026, and the Modules 2 to 7 searches on 11 August 2026. The upper coverage limit is the date each search was executed. Although the queries use `"3000"[Date - Publication]` as the upper publication-date boundary, only records available in PubMed on the search date could be retrieved, so the search date set the effective upper limit. For every search, the number of records obtained matched the number PubMed reported, with none missing. The disease terms and date limits differ across modules because each search targeted a different topic; the topics are given in the Topic column of **Supplementary Tables S1** and **S2**.

Deduplication was applied within each search and then across searches in the order shown, by PMID, then by DOI, then by exact title. The count of new unique records therefore depends on the order in which searches were processed; the spatial search overlapped substantially with the single-cell search run before it.

Module 1 was run first, and the 3,408 records remaining after cross-search deduplication formed the screening universe for that module: all were screened by title and abstract against the prespecified scope, and records that could not be resolved at that stage were carried to full-text review rather than excluded. This produced a shortlist of 73 records, of which 66 were obtained and extracted for detailed evidence assessment.

For Modules 2 to 7, records identified from the PubMed publication-type field as retracted publications, case reports, editorials, comments, corrections, preprints, or book chapters were removed before prioritization. The remaining records were ranked by relevance to the prespecified topics; ranking set the order in which records were read and was not an eligibility criterion. A defined number of the highest-ranked records from each module was then screened manually by title, with the abstract consulted where the title alone was insufficient. Screening was targeted rather than exhaustive, as is appropriate to a purposive narrative review.

Studies were eligible when they used an interpretable design and reported a result relevant to a prespecified topic in HCC or iCCA. Evidence from other tumor types was eligible only where it supplied a directly relevant mechanism or a narrowly bounded therapeutic rationale, and its non-hepatic context was stated explicitly wherever it is cited. Case reports, non-English reports, unvalidated in silico predictions, and correlation-only analyses without mechanistic or translational interpretation were excluded.

Among eligible records, randomized trials and mature prospective studies were prioritized for claims about clinical efficacy, and human spatial or single-cell studies and functionally validated experiments were prioritized for biological claims. Studies were also retained when they showed heterogeneity, disagreement between reports, or a limitation of the prevailing interpretation.

Records that could not be resolved at screening were carried forward to full-text assessment rather than excluded.

One conference abstract was retained: the EMERALD-3 report presented at the 2026 American Society of Clinical Oncology Annual Meeting, for which no peer-reviewed full publication was available at the final search date. It is identified as a conference abstract wherever it is cited.

No formal risk-of-bias assessment and no quantitative synthesis were performed.

Supplementary Table S1. Module 1 search: single-cell, spatial, and stromal architecture. All unique records were screened by title and abstract; records that could not be resolved at that stage were carried to full-text review rather than excluded. Searches were run on 10 August 2026 and covered records published from 1 January 2016 through the search date.

Search ID	Topic	Records retrieved	Unique within search	New unique after cross-search deduplication	Shortlisted for full-text assessment	Full texts obtained and extracted
M1A-PM-01	Single-cell and single-nucleus profiling	1,991	1,987	1,987	—	—
M1B-PM-01	Spatial transcriptomics and multiplex imaging	492	490	172	—	—
M1C-PM-01	Fibroblasts, stellate cells, desmoplasia, and extracellular matrix	1,523	1,519	1,249	—	—
Module 1 total		4,006	3,996	3,408	73	66

M1A-PM-01—single-cell and single-nucleus profiling

(("Carcinoma, Hepatocellular"[Mesh] OR hepatocellular carcinoma[Title/Abstract] OR HCC[Title/Abstract]) OR ("Cholangiocarcinoma"[Mesh] OR intrahepatic cholangiocarcinoma[Title/Abstract] OR intrahepatic cholangiocarcinomas[Title/Abstract] OR iCCA[Title/Abstract])) AND ("single-cell"[Title/Abstract] OR "single cell"[Title/Abstract] OR "single-nucleus"[Title/Abstract] OR "single nucleus"[Title/Abstract] OR scRNA-seq[Title/Abstract] OR snRNA-seq[Title/Abstract]) AND ("2016/01/01"[Date - Publication] : "3000"[Date - Publication]) AND English[Language]

M1B-PM-01 — spatial transcriptomics and multiplex imaging

(("Carcinoma, Hepatocellular"[Mesh] OR hepatocellular carcinoma[Title/Abstract] OR HCC[Title/Abstract]) OR ("Cholangiocarcinoma"[Mesh] OR intrahepatic cholangiocarcinoma[Title/Abstract] OR intrahepatic cholangiocarcinomas[Title/Abstract] OR iCCA[Title/Abstract])) AND ("spatial transcriptom*"[Title/Abstract] OR "spatially resolved"[Title/Abstract] OR "spatial proteomic*"[Title/Abstract] OR "multiplex immunofluorescence"[Title/Abstract] OR "multiplex imaging"[Title/Abstract] OR "imaging mass cytometry"[Title/Abstract] OR "multiplex ion beam imaging"[Title/Abstract] OR MIBI[Title/Abstract]) AND ("2016/01/01"[Date - Publication] : "3000"[Date - Publication]) AND English[Language]

M1C-PM-01 — fibroblasts, stellate cells, desmoplasia, and extracellular matrix

(("Carcinoma, Hepatocellular"[Mesh] OR hepatocellular carcinoma[Title/Abstract] OR HCC[Title/Abstract]) OR ("Cholangiocarcinoma"[Mesh] OR intrahepatic cholangiocarcinoma[Title/Abstract] OR intrahepatic cholangiocarcinomas[Title/Abstract] OR iCCA[Title/Abstract])) AND ("cancer-associated fibroblast*"[Title/Abstract] OR "tumor-associated fibroblast*"[Title/Abstract] OR "tumour-associated fibroblast*"[Title/Abstract] OR "hepatic stellate cell*"[Title/Abstract] OR myofibroblast*[Title/Abstract] OR desmoplas*[Title/Abstract] OR stromal[Title/Abstract] OR "extracellular matrix"[Title/Abstract] OR "matrix remodeling"[Title/Abstract] OR "matrix remodelling"[Title/Abstract]) AND (architecture[Title/Abstract] OR heterogeneity[Title/Abstract] OR microenvironment[Title/Abstract] OR spatial[Title/Abstract] OR transcriptom*[Title/Abstract] OR proteom*[Title/Abstract] OR interaction*[Title/Abstract]) AND ("2016/01/01"[Date - Publication] : "3000"[Date - Publication]) AND English[Language]

Supplementary Table S2. Modules 2 to 7: targeted searches for immune populations, tumor-intrinsic programs, treatment, and natural products. Records were ranked by relevance to the prespecified topics, and a defined number of the highest-ranked records from each module was screened by title. Searches were run on 11 August 2026.

Search ID	Topic	Coverage interval	Records retrieved	Unique within search	New unique after cross-module deduplication	Records manually screened	Candidate studies carried forward	Studies selected for use
M2-PM-01	Additional immune populations	1 January 2016 to search date	3,227	3,227	3,227	148	13	9
M3-PM-01	Tumor-intrinsic programs	1 January 2016 to search date	2,512	2,512	2,276	125	10	4
M4-PM-01	Nicotinamide N-methyltransferase in liver cancer	Database inception to search date	22	22	22	22	2	0
M4-PM-02	Nicotinamide N-methyltransferase inhibitors and antitumor immunity	Database inception to search date	129	129	124	122	2	1
M5-PM-01	Chemo-immunotherapy in biliary tract cancer	Database inception to search date	296	296	257	118	5	3
M6-PM-01	Emerging immunotherapies and engineered immune cells	1 January 2016 to search date	959	959	696	105	10	5
M7-PM-01	Natural products and traditional Chinese medicine	1 January 2016 to search date	554	554	449	113	3	1
Modules 2-7 total			7,699	7,699	7,051	753	45	23

M2-PM-01—additional immune populations

(("Carcinoma, Hepatocellular"[Mesh] OR hepatocellular carcinoma[Title/Abstract] OR HCC[Title/Abstract]) OR ("Cholangiocarcinoma"[Mesh] OR intrahepatic cholangiocarcinoma[Title/Abstract] OR intrahepatic cholangiocarcinomas[Title/Abstract] OR iCCA[Title/Abstract])) AND ("tumor-associated neutrophil*"[Title/Abstract] OR "tumour-associated neutrophil*"[Title/Abstract] OR neutrophil*[Title/Abstract] OR "myeloid-derived suppressor cell*"[Title/Abstract] OR MDSC[Title/Abstract] OR MDSCs[Title/Abstract] OR "dendritic cell*"[Title/Abstract] OR "natural killer cell*"[Title/Abstract] OR "NK cell*"[Title/Abstract] OR "natural killer T cell*"[Title/Abstract] OR "NKT cell*"[Title/Abstract] OR "mucosal-associated invariant T"[Title/Abstract] OR MAIT[Title/Abstract] OR "gamma delta T"[Title/Abstract] OR "gamma-delta T"[Title/Abstract] OR "γδ T"[Title/Abstract] OR "B cell*"[Title/Abstract] OR "tertiary lymphoid structure*"[Title/Abstract] OR TLS[Title/Abstract]) AND (microenvironment[Title/Abstract] OR immune[Title/Abstract] OR immun*[Title/Abstract] OR spatial[Title/Abstract]) AND ("2016/01/01"[Date - Publication] : "3000"[Date - Publication]) AND English[Language]

M3-PM-01 — tumor-intrinsic programs

(("Carcinoma, Hepatocellular"[Mesh] OR hepatocellular carcinoma[Title/Abstract] OR HCC[Title/Abstract]) OR ("Cholangiocarcinoma"[Mesh] OR intrahepatic cholangiocarcinoma[Title/Abstract] OR intrahepatic cholangiocarcinomas[Title/Abstract] OR iCCA[Title/Abstract])) AND (CTNNB1[Title/Abstract] OR "beta-catenin"[Title/Abstract] OR "β-catenin"[Title/Abstract] OR TP53[Title/Abstract] OR MYC[Title/Abstract] OR "TGF-beta"[Title/Abstract] OR "TGF-β"[Title/Abstract] OR FGFR2[Title/Abstract] OR KRAS[Title/Abstract] OR BAP1[Title/Abstract] OR IDH1[Title/Abstract] OR IDH2[Title/Abstract] OR "isocitrate dehydrogenase"[Title/Abstract]) AND ("tumor

microenvironment"[Title/Abstract] OR "tumour microenvironment"[Title/Abstract] OR immune[Title/Abstract] OR immun*[Title/Abstract] OR stromal[Title/Abstract] OR fibroblast*[Title/Abstract] OR macrophage*[Title/Abstract] OR lymphocyte*[Title/Abstract] OR exclusion[Title/Abstract]) AND ("2016/01/01"[Date - Publication] : "3000"[Date - Publication]) AND English[Language]

M4-PM-01 — nicotinamide N-methyltransferase in liver cancer

("Carcinoma, Hepatocellular"[Mesh] OR hepatocellular carcinoma[Title/Abstract] OR HCC[Title/Abstract] OR liver cancer[Title/Abstract]) AND ("nicotinamide N-methyltransferase"[Title/Abstract] OR NNMT[Title/Abstract]) AND English[Language]

M4-PM-02 — nicotinamide N-methyltransferase inhibitors and antitumor immunity

("nicotinamide N-methyltransferase"[Title/Abstract] OR NNMT[Title/Abstract]) AND (inhibitor*[Title/Abstract] OR pharmacolog*[Title/Abstract] OR therapeutic*[Title/Abstract] OR "immune checkpoint"[Title/Abstract] OR "PD-1"[Title/Abstract] OR "PD-L1"[Title/Abstract]) AND cancer[Title/Abstract] AND English[Language]

M5-PM-01 — chemo-immunotherapy in biliary tract cancer

("Cholangiocarcinoma"[Mesh] OR intrahepatic cholangiocarcinoma[Title/Abstract] OR iCCA[Title/Abstract] OR biliary tract cancer[Title/Abstract] OR biliary tract cancers[Title/Abstract]) AND ((gemcitabine[Title/Abstract] AND cisplatin[Title/Abstract]) AND (durvalumab[Title/Abstract] OR pembrolizumab[Title/Abstract] OR "immune checkpoint"[Title/Abstract] OR immunotherapy[Title/Abstract]) OR TOPAZ-1[Title/Abstract] OR KEYNOTE-966[Title/Abstract]) AND English[Language]

M6-PM-01—emerging immunotherapies and engineered immune cells

(("Carcinoma, Hepatocellular"[Mesh] OR hepatocellular carcinoma[Title/Abstract] OR HCC[Title/Abstract]) OR ("Cholangiocarcinoma"[Mesh] OR intrahepatic cholangiocarcinoma[Title/Abstract] OR intrahepatic cholangiocarcinomas[Title/Abstract] OR iCCA[Title/Abstract] OR biliary tract cancer[Title/Abstract])) AND (TIM-3[Title/Abstract] OR TIM3[Title/Abstract] OR HAVCR2[Title/Abstract] OR CTLA-4[Title/Abstract] OR CTLA4[Title/Abstract] OR "cancer vaccine*"[Title/Abstract] OR "tumor vaccine*"[Title/Abstract] OR "tumour vaccine*"[Title/Abstract] OR "CAR-T"[Title/Abstract] OR "chimeric antigen receptor T"[Title/Abstract] OR "CAR-NK"[Title/Abstract] OR "chimeric antigen receptor natural killer"[Title/Abstract] OR "CAR macrophage*"[Title/Abstract] OR "CAR-M"[Title/Abstract] OR "chimeric antigen receptor macrophage*"[Title/Abstract]) AND (therapy[Title/Abstract] OR treatment[Title/Abstract] OR trial[Title/Abstract] OR preclinical[Title/Abstract] OR microenvironment[Title/Abstract]) AND ("2016/01/01"[Date - Publication] : "3000"[Date - Publication]) AND English[Language]

M7-PM-01 — natural products and traditional Chinese medicine

("Carcinoma, Hepatocellular"[Mesh] OR hepatocellular carcinoma[Title/Abstract] OR HCC[Title/Abstract] OR liver cancer[Title/Abstract]) AND ("Medicine, Chinese Traditional"[Mesh] OR "traditional Chinese medicine"[Title/Abstract] OR TCM[Title/Abstract] OR ginsenoside*[Title/Abstract] OR curcumin[Title/Abstract] OR matrine[Title/Abstract] OR "astragalus polysaccharide*"[Title/Abstract] OR "herbal medicine"[Title/Abstract] OR "natural product*"[Title/Abstract]) AND ("tumor microenvironment"[Title/Abstract] OR "tumour microenvironment"[Title/Abstract] OR immune[Title/Abstract] OR immun*[Title/Abstract] OR macrophage*[Title/Abstract] OR "dendritic cell*"[Title/Abstract] OR "CD8"[Title/Abstract] OR "MHC-I"[Title/Abstract] OR "PD-1"[Title/Abstract] OR "PD-L1"[Title/Abstract] OR stromal[Title/Abstract]) AND ("2016/01/01"[Date - Publication] : "3000"[Date - Publication]) AND English[Language]
